# First person – Qingting Hu

**DOI:** 10.1242/bio.045484

**Published:** 2019-07-15

## Abstract

First Person is a series of interviews with the first authors of a selection of papers published in Biology Open helping early-career researchers promote themselves alongside their papers. Qingting Hu is first author on ‘Role of ALDH1A1 and HTRA2 expression in CCL2/CCR2-mediated breast cancer cell growth and invasion’, published in BIO. Qingting conducted the research described in this article while a Research Technician in Dr Nikki Cheng's lab at University of Kansas Medical Center, USA. She is now a Research Technician in the lab of Dr. Tonks Nicholas at Cold Spring Harbor Laboratory, USA, investigating downstream signaling mechanism of CCL2/CCR2 pathway in breast cancer progression.


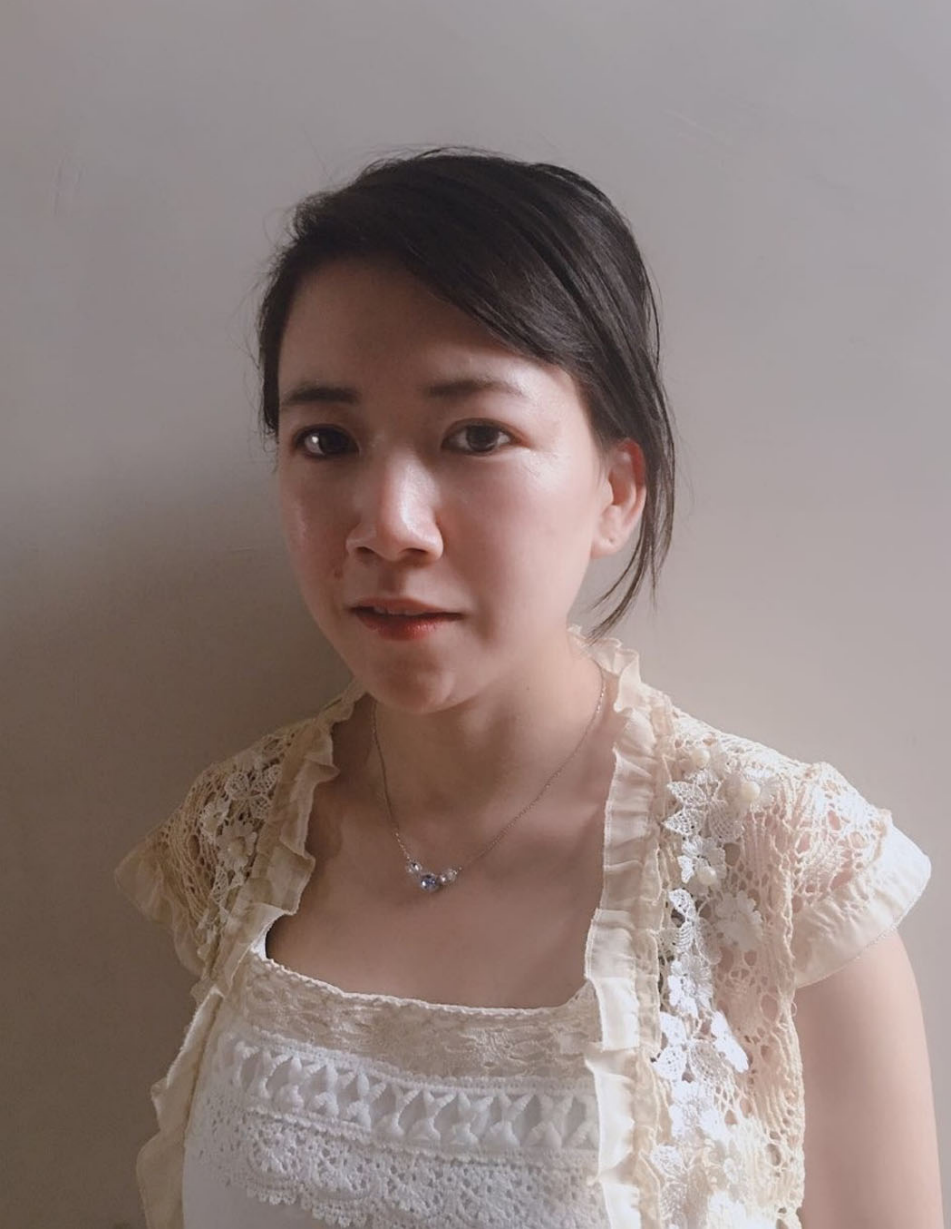


**Qingting Hu**

**What is your scientific background and the general focus of your lab?**

I have a master's degree and an M.D. During the current study, I was working in Dr Cheng's laboratory as an exchange scholar from China. The major focus of Dr Cheng's laboratory is to study the molecular interactions between cancer cells and fibroblasts in breast cancer progression, and her lab has discovered that fibroblasts secrete high levels of the chemokine CCL2, and that CCL2 signals to breast cancer cells to promote breast cancer invasion through CCR2-dependent mechanisms. My research focus was to understand the molecular mechanisms of CCL2/CCR2 signaling in early stage breast cancer models.

**How would you explain the main findings of your paper to non-scientific family and friends?**

Our studies have discovered two molecules called ALDH1A1 and HTRA2 that are important for breast cancer growth and invasion. Expression and activity of these molecules are regulated by a factor called CCL2, which is normally important for immune responses.

“Our studies have discovered two molecules called ALDH1A1 and HTRA2 that are important for breast cancer growth and invasion.”

**What are the potential implications of these results for your field of research?**

Our detailed description on the molecular mechanisms of CCL2/CCR2 signaling in breast cancer cell growth and invasion can be used to identify biomarkers in order to better predict DCIS progression. These biomarkers could also be potential therapeutic targets to prevent or treat invasive breast cancer.

**What has surprised you the most while conducting your research?**

The most surprising results came in the beginning, when we treated two breast cancer cell lines (DCIS.com and SUM225) with recombinant CCL2, but only observed a responsive phenotype in DCIS.com cells. We were initially puzzled, but we reasoned that it was due to different expression levels of CCR2 receptors in those two cell lines. We confirmed this by flow cytometry analysis and that led to subsequent CCR2 overexpression and knockout/knockdown studies.

**What, in your opinion, are some of the greatest achievements in your field and how has this influenced your research?**

One advancement of the cancer research field is the development of 3D culture models to mimic tumor tissues. This provides a great advantage compared to traditional 2D cell culture, as it more closely mimics the structural features of breast tissues. That is why we used 3D culture models to characterize the mechanisms of CCL2/CCR2 signaling in breast cancer cells.

“One advancement of the cancer research field is the development of 3D culture models to mimic tumor tissues.”

**What changes do you think could improve the professional lives of early-career scientists?**

We early-career scientists often learn from making errors and mistakes. An encouraging mentor and good guidance are important. I was lucky to have such a mentor.

**Figure:**
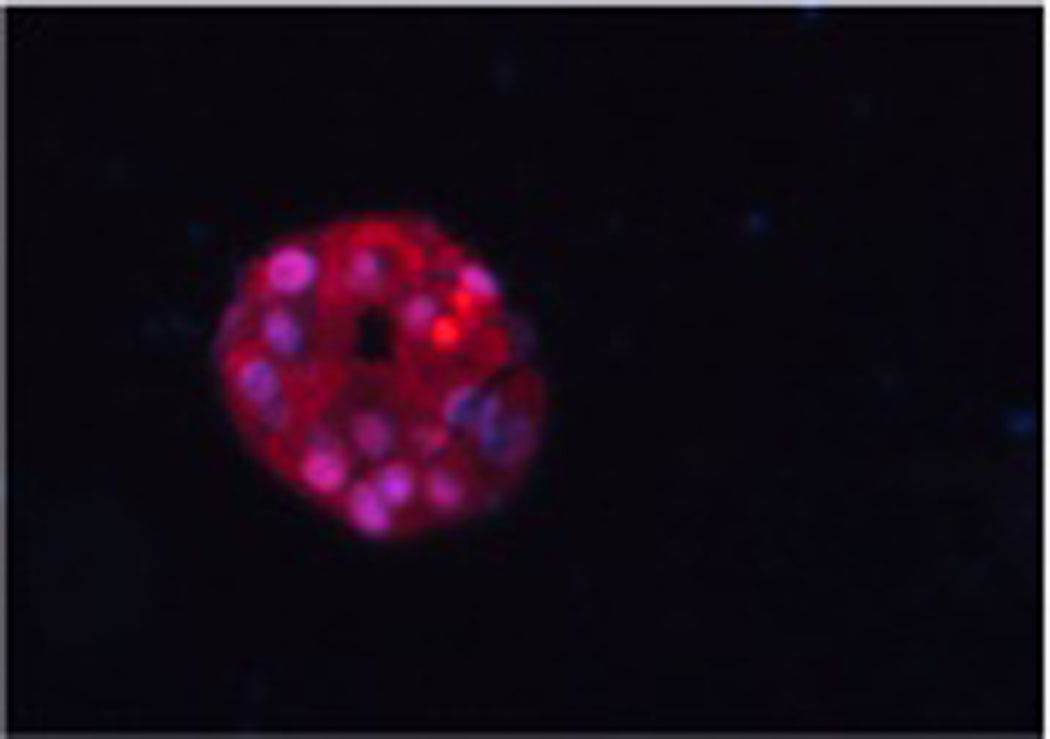
**Sum225 culture in 3D.**

**What's next for you?**

Apply for a Ph.D. program for further training in translational research.
